# Diversity-guided Lamarckian random drift particle swarm optimization for flexible ligand docking

**DOI:** 10.1186/s12859-020-03630-2

**Published:** 2020-07-06

**Authors:** Chao Li, Jun Sun, Vasile Palade

**Affiliations:** 1 Key Laboratory of Advanced Process Control for Light Industry (Ministry of Education), No. 1800, Lihu Avenue, Wuxi, Jiangsu 214122 PR China; 2 Faculty of Engineering and Computing, Coventry University, Priory Street, Coventry, CV1 5FB UK

**Keywords:** Flexible ligand docking, Search algorithms, Random drift particle swarm optimization, Diversity control strategy, Solis and Wets local search, Autodock software

## Abstract

**Background:**

Protein-ligand docking has emerged as a particularly important tool in drug design and development, and flexible ligand docking is a widely used method for docking simulations. Many docking software packages can simulate flexible ligand docking, and among them, Autodock is widely used. Focusing on the search algorithm used in Autodock, many new optimization approaches have been proposed over the last few decades. However, despite the large number of alternatives, we are still lacking a search method with high robustness and high performance.

**Results:**

In this paper, in conjunction with the popular Autodock software, a novel hybrid version of the random drift particle swarm optimization (RDPSO) algorithm, called diversity-guided Lamarckian RDPSO (DGLRDPSO), is proposed to further enhance the performance and robustness of flexible ligand docking. In this algorithm, a novel two-phase diversity control (2PDC) strategy and an efficient local search strategy are used to improve the search ability and robustness of the RDPSO algorithm. By using the PDBbind coreset v.2016 and 24 complexes with apo-structures, the DGLRDPSO algorithm is compared with the Lamarckian genetic algorithm (LGA), Lamarckian particle swarm optimization (LPSO) and Lamarckian random drift particle swarm optimization (LRDPSO). The experimental results show that the 2PDC strategy is able to enhance the robustness and search performance of the proposed algorithm; for test cases with different numbers of torsions, the DGLRDPSO outperforms the LGA and LPSO in finding both low-energy and small-RMSD docking conformations with high robustness in most cases.

**Conclusion:**

The DGLRDPSO algorithm has good search performance and a high possibility of finding a conformation with both a low binding free energy and a small RMSD. Among all the tested algorithms, DGLRDPSO has the best robustness in solving both holo- and apo-structure docking problems with different numbers of torsions, which indicates that the proposed algorithm is a reliable choice for the flexible ligand docking in Autodock software.

## Background

Protein-ligand docking methods are of the utmost importance and have been widely used in drug discovery and other academic research areas [1]. Generally, these methods aim to predict the experimental binding modes and affinities of small molecules within the binding site of particular receptor targets. They offer a relatively fast and economic alternative to the standard experimental techniques [2]. Considering the efficiency and accuracy, flexible ligand docking is the most conventionally adopted method in protein-ligand docking [3]. It can be solved by exploring the suitable translations, orientations and conformations of a ligand, while the protein is considered a rigid object. Many docking software packages can simulate flexible ligand docking by using suitable search methods and scoring functions [4], among which Autodock [5] is widely used. It is a versatile protein-ligand docking program with good accuracy and high versatility, making it a very popular choice for drug developers. In the latest version, Autodock uses a semi-empirical energy function as its scoring function to estimate the free energy change upon binding, and it offers a variety of search algorithms, mainly including a Monte Carlo simulated annealing algorithm and a genetic algorithm with the Solis and Wets local search method, which is also called the Lamarckian genetic algorithm (LGA) [5].

Many new optimization approaches have been designed based on the Autodock software, and a large part of them focus on the improvement of search algorithms. SODOCK [3] is a sophisticated protein-ligand docking program based on Autodock 3.05, which uses an adaptation of the particle swarm optimization (PSO) combined with the neighbourhood topology and the Solis and Wets local search as its search algorithm. This search algorithm has already been integrated into the latest version of Autodock, named the “Lamarckian PSO (LPSO)”. PSO@AUTODOCK [6] includes two variants of the PSO algorithm (varCPSO and varCPSO-Ls) designed for the rapid docking of highly flexible ligands. A more recent study integrated Autodock with jMetalCpp [7], constructing an optimization framework that provides both single- and multi-objective algorithms that can be used to effectively solve docking problems. Other versions of Autodock [8–10] also achieved some improvements. However, despite the large number of alternatives, we are still far from a high-performance docking program. In terms of the search algorithms in Autodock, regardless of whether the flexible ligand docking problem has a small or large number of search dimensions, it remains a challenging task to efficiently optimize the binding free energy with high robustness and to find a final result close to the theoretical minimum.

To further enhance the performance and robustness of the search algorithms used in the Autodock software for flexible ligand docking, in this paper, based on the random drift particle swarm optimization (RDPSO) algorithm [11], we propose a hybrid search method called the diversity-guided Lamarckian RDPSO (DGLRDPSO). In DGLRDPSO, a novel two-phase diversity control (2PDC) strategy and an efficient variant of the Solis and Wets [12] method are used to enhance the search ability and robustness of the search algorithm. By using all 285 test cases in PDBbind coreset v.2016 [13] and 24 crystallographic complexes with known ligands docked to the active-site apo structures of thrombin [14, 15], we made a performance comparison among LGA, LPSO (the search algorithm used in SODOCK [3]), DGLRDPSO, and Lamarckian RDPSO (LRDPSO, the canonical RDPSO combined with the Solis and Wets local search). A docking performance comparison was made among the first three algorithms.

The results of the performance comparison reveal that the 2PDC strategy improves the performance and robustness of the DGLRDPSO so that its search performance is superior to all the other compared algorithms in most cases. For the docking performance, the DGLRDPSO also outperforms the LGA and LPSO for both holo- and apo-structure docking problems in most cases, especially for the cases with highly flexible ligands, with better robustness than the other algorithms in terms of the number of torsions. Therefore, the proposed algorithm is expected to be a good choice for flexible ligand docking.

## Methods

### Particle swarm optimization

Particle swarm optimization (PSO) is an important metaheuristic algorithm inspired by the social behaviour of bird flocks and was first proposed by Eberhart and Kennedy [16]. It is an easy and inexpensive method for performing the optimization tasks of non-continuous, complex and global optimization problems.

In a PSO with *M* individual particles, the current position vector and the velocity vector of particle *i* (1 ≤ *i* ≤ *M*) at the *n*^*th*^ iteration are represented by ${X}_{i,n}=\left({X}_{i,n}^1,{X}_{i,n}^2,\cdots, {X}_{i,n}^N\right)$ and ${V}_{i,n}=\left({V}_{i,n}^1,{V}_{i,n}^2,\cdots, {V}_{i,n}^N\right)$, respectively, for an *N*-dimensional optimization problem. The particle moves according to the following equations:

1 $$ {V}_{i,n+1}^j={V}_{i,n}^j+{c}_1{r}_{i,n}^j\left({P}_{i,n}^j-{X}_{i,n}^j\right)+{c}_2{R}_{i,n}^j\left({G}_n^j-{X}_{i,n}^j\right) $$

2 $$ {X}_{i,n+1}^j={X}_{i,n}^j+{V}_{i,n+1}^j $$

where *i* = 1, 2⋯, *M*; *j* = 1, 2⋯, *N*; and *c*_1_ and *c*_2_ are known as the acceleration coefficients. Vector ${P}_{i,n}=\left({P}_{i,n}^1,{P}_{i,n}^2,\cdots, {P}_{i,n}^N\right)$ is the previous best position of particle *i*, called the personal best (pbest) position, and vector ${G}_n=\left({G}_n^1,{G}_n^2,\cdots, {G}_n^N\right)$ is the best position among all the *pbest* positions in the population and is called the global best (gbest) position. The pbest positions are updated by comparing the fitness values of the particle’s current position and its own pbest position. The parameters ${r}_{i,n}^j$ and ${R}_{i,n}^j$ are sequences of two different random numbers distributed uniformly in the (0, 1) interval, which is denoted by ${r}_{i,n}^j,{R}_{i,n}^j\sim U\left(0,1\right)$. The velocity of a particle should be restricted in the interval [−*V*_*max*_, *V*_*max*_] as follows:

3 $$ {\displaystyle \begin{array}{c} If\kern0.5em {V}_{i,n}^j>{V}_{max},{thenV}_{i,n}^j={V}_{max}\\ {} If\kern0.5em {V}_{i,n}^j<-{V}_{max},{thenV}_{i,n}^j=-{V}_{max}\end{array}} $$

### Random drift particle swarm optimization

The RDPSO is a variant of the PSO motivated by the trajectory analysis of the canonical PSO in [17] and the free electron model in the metal conductors placed in an external electric field [18]. In RDPSO, it was assumed that the particle behaves similar to an electron moving in a metal conductor in an external electric field. The movement of the particle is thus the superposition of the thermal and drift motions corresponding to the random velocity and the drift velocity, respectively. The RDPSO algorithm has a better performance than canonical PSO in most cases, as shown in [11].

The random velocity component ${VR}_{i,n+1}^j$ implements the global search, following the Maxwell velocity distribution law, and thus is expressed as

4 $$ {VR}_{i,n+1}^j=\alpha \mid {C}_n^j-{X}_{i,n}^j\mid {\varphi}_{i,n+1}^j $$

where *α* > 0 is a parameter called the thermal coefficient, *C*_*n*_ is the mean best (mbest) position defined by the mean of the pbest positions of all the particles, and ${\varphi}_{i,n+1}^j$ is the sequence of the random numbers subject to a standard normal distribution, i.e., ${\varphi}_{i,n+1}^j\sim N\left(0,1\right)$.

The drift velocity component ${VD}_{i,n+1}^j$ is used to achieve the local search of the particle. It is given by

5 $$ {VD}_{i,n+1}^j=\beta \left({p}_{i,n}^j-{X}_{i,n}^{\mathrm{j}}\right) $$

where *β* > 0 is another algorithmic parameter called the drift coefficient. ${p}_{i,n}=\left({p}_{i,n}^1,{p}_{i,n}^2,\cdots, {p}_{i,n}^N\right)$ is the local focus of particle *i* in the canonical PSO and is expressed in [17]:

6 $$ {p}_{i,n}^j={\gamma}_{i,n}^j{P}_{i,n}^j+\left(1-{\gamma}_{i,n}^j\right){G}_n^j,{\gamma}_{i,n}^j\sim U\left(0,1\right) $$

Thus, the update equations for the velocity and position vectors of the particle in the RDPSO algorithm are given by

7 $$ {V}_{i,n+1}^j={VR}_{i,n+1}^j+{VD}_{i,n+1}^j=\alpha \mid {C}_n^j-{X}_{i,n}^j\mid {\varphi}_{i,n+1}^j+\beta \left({p}_{i,n}^j-{X}_{i,n}^j\right) $$

8 $$ {X}_{i,n+1}^j={X}_{i,n}^j+{V}_{i,n+1}^j $$

where the value of ${V}_{i,n}^j$ should also be restricted within the interval [−*V*_*max*_, *V*_*max*_], following eq. (3). It is recommended in [11] that when *α* decreases linearly from 0.9 to 0.3 and *β* = 1.45, the algorithm can obtain generally better performance than the algorithms with other parameter configurations. In this paper, RDPSO with this setting of *α* and *β* is called the canonical RDPSO.

### Two-phased diversity control strategy

In our previous work, we used the RDPSO algorithm directly in flexible ligand docking, but it was found that in many test cases, the algorithm encountered premature convergence and became trapped a local optima frequently, leading to unsatisfactory results. To avoid premature convergence in the RDPSO algorithm, there should be good balance between the global search and local search of the particle. Particularly, in the later stage of the search process, the particle generally has a weak global search ability. Thus, in this paper, we propose a strategy of controlling the diversity of the swarm in RDPSO to ensure that the particle has a constant balance between its local and global search. As in [19], the swarm diversity is measured by the average distance from the average point of the swarm, that is,

9 $$ D\left({X}_n\right)=\frac{1}{M\bullet A}{\sum}_{i=1}^M{\left[{\sum}_{j=1}^N{\left[{X}_{i,n}^j-\overline{X_n^j}\right]}^2\right]}^{1/2}=\frac{1}{M\bullet A}{\sum}_{i=1}^M\left|{X}_{i,n}-\overline{X_n}\right| $$

where *M* is the number of particles in the swarm, *N* is the dimensionality, and *A* is the diagonal length of the search space, which represents the size of the search area. $\overline{X_n^j}$ is the *j*^th^ dimension of the mean of all particle positions.

By this definition, *D*(*X*_*n*_) measures the dispersion of the distribution of all the particles’ positions in the search space and is known as the swarm diversity [19]. A larger *D*(*X*_*n*_) generally corresponds to the relatively more dispersed distribution of the particles and implies a stronger global search ability of the particle swarm. On the other hand, a smaller *D*(*X*_*n*_) represents a more aggregated distribution of the particle and therefore means a stronger local search ability of the swarm. For the RDPSO and other PSO algorithms, the diversity is relatively large during the early stage of the search process so that the global search ability of the particle swarm is stronger. At this point in the process, the rapid decrease in diversity is desirable to balance the exploration and exploitation of the swarm. However, during the later stage of the search process, the diversity may decline to such a small scale that the particles aggregate into a small area and further global search becomes impossible, and premature convergence occurs as a result if the global optimal solution is not in this area. Therefore, to avoid premature convergence and thus improve the search performance of the algorithm, applying a diversity control strategy is an effective way to control the variation of the swarm diversity during the search process. In particular, the controlling strategy for swarm diversity should allow for a gradual decrease in diversity so that a certain level of diversity is maintained to ensure that the swarm has animation for further search.

With the above justification, in this paper, we propose a diversity control strategy for RDPSO to improve the algorithmic performance. In this improved RDPSO, the swarm diversity is measured at each iteration and is controlled according to the following strategy.

#### A. Lower bound of the diversity values

First, we propose a strategy of linearly decreasing the lower bound of the diversity values by

10 $$ {DL}_n=\left(1-n/n\_\mathit{\max}\right)\ast \left({DL}_1-{DL}_{n\_\mathit{\max}}\right)+{DL}_{n\_\mathit{\max}} $$

where *n* represents the *n*^*th*^ iteration of the algorithm and *n* _ *max* is the maximum number of iterations. Empirically, *DL*_1_ and *DL*_*n* _ *max*_ are set to be 0.75 ∗ *D*(*X*_1_) and 0.002 ∗ *D*(*X*_1_), respectively. Both of these parameters are associated with the diversity value of first iteration *D*(*X*_1_) since the positions of the particles are set randomly at the beginning of the search. The linear decreasing lower bound of the diversity makes the particles’ search scope reduce smoothly, searching more globally at the beginning, to avoid a significant drop in swarm diversity, and searching more locally at the end to find a result with high solution precision.

#### B. Normal convergence of particles

If the diversity value is higher than the lower bound and the algorithm is not in the divergence phase, which is described in subsection C, the particles converge normally, following eqs. (7) and (8), where the values of *α* and *β* are selected as those in the canonical RDPSO. The value of *V*_*max*_ is set as:

11 $$ {V}_{max}^j=\left({X}_{max}^j-{X}_{min}^j\right)/2 $$

where ${X}_{max}^j$ and ${X}_{min}^j$ are the maximum and minimum values that the particles can reach in the *j*^*th*^ dimension, respectively.

#### C. Divergence of particles

When the swarm diversity becomes lower than the lower bound, the particles stop converging to prevent the diversity from dropping constantly. To this end, the particles are forced to diverge until the swarm diversity increases to *DU*_*n*_, the upper bound of the diversity fixed at 0.95 ∗ *D*(*X*_1_). When the diversity reaches *DU*_*n*_, the particles can be distributed over the search space, and the swarm is then converted to the mode of normal convergence with the newly enhanced global search ability.

For the purpose of making the particle swarm diverge, we can set *α* and *β* to high values as follows to lead the particles to escape from both the *gbest* and *pbest* positions:

12 $$ Dr\left({X}_n\right)=D\left({X}_n\right)/D\left({X}_1\right) $$

13 $$ \alpha ={\alpha}_0/{\left( Dr\left({X}_n\right)\right)}^{c1} $$

14 $$ \beta ={\beta}_0/{\left( Dr\left({X}_n\right)\right)}^{c2} $$

where *Dr*(*X*_*n*_) measures the degree of dispersion of the particle swarm, with *D*(*X*_1_) used as a baseline for the diversity. It should be noted that during the divergence process, the particles have little chance to find a better solution; thus, the main purpose is to disperse the particles so that the particles can search more globally in the convergence stage. As such, in equations (13) and (14), the reciprocal form of *Dr*(*X*_*n*_) is used to make *α* and *β* increase dramatically while decreasing the swarm diversity, and as a result, the swarm diversity can increase to a high value after only a few iterations. Here, we set *α*_0_ = 9 and *β*_0_ = 3, where *c*1 and *c*2 are employed to adjust the changing amplitudes of *α* and *β*. Our preliminary experiments showed that setting *c*1 = 1 and *c*2 = 0.7 can help the diversity increase to the upper bound very quickly.

#### D. Accelerated convergence of particles

The preliminary experiments on some benchmark functions showed that the diversity may not be able to drop to the lower bound since the particles probably converge too slowly. In this case, the final solutions obtained may be poor due to the weak local search ability of the algorithm. To address this problem, we design an acceleration mechanism in which the pbest position of the particle *P*_*i*, *n*_ is set to be its local attractor *p*_*i*, *n*_ if the swarm diversity fails to reach the lower bound after *accr* ∗ *n* _ *max* iterations in the current convergence phase. Here, *accr* is generally no less than 0.003. Such an acceleration mechanism can force the pbest positions to move closer to the gbest position, making the particles search in a smaller scope and thus decreasing the swarm diversity.

The above diversity control strategy is called the two-phased diversity control (2PDC) strategy, as the iterative process of the RDPSO has been divided into the convergence phase and the divergence phase. With such a strategy, premature convergence can be effectively avoided, and a good balance between the global search and local search abilities can be achieved.

### Solis and Wets local search method and the hybrid search algorithm

To further increase the possibility of finding the global optimal solution, the Solis and Wets local search method is employed in this work [12]. The Solis and Wets method is a stochastic heuristic for continuous parameter spaces, which introduces a probabilistic element. Its primal purpose is the local optimization of functions that do not provide gradient information [12]. Basically, the local optimization starts by exploring a random direction in the search space and generally follows this direction with random movements as long as the fitness function continues to improve. The continued improvements lead to an expansion of the random search steps, whereas the continued failings narrow the search [5]. In our experiments, the maximum number of iterations in the Solis and Wets method is set to 300; the maximum number of consecutive successes or failures before doubling or halving the local search step size is set to 4; and the lower bound of the step size, which is also the termination criterion for the local search, is set to 0.01. In the hybrid algorithm, this local search method is only applied to the best particle in each iteration. For a more specific procedure of the algorithm, one can refer to [12].

As the local search method is based on the Lamarck evolution mechanism, the hybrid algorithm combining the RDPSO, 2PDC strategy and the Solis and Wets method is named the diversity-guided Lamarckian RDPSO (DGLRDPSO) algorithm, which is implemented in the Autodock software in this work. The procedure of the algorithm is outlined below.

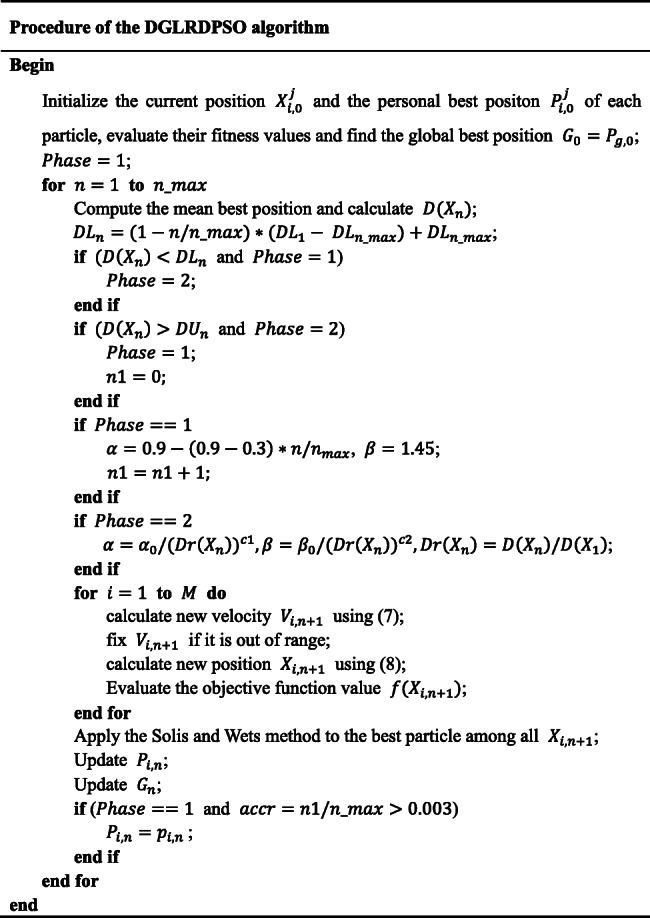



### Data set

To empirically evaluate the effectiveness of the DGLRDPSO algorithm in docking, the PDBbind coreset v.2016 (http://www.pdbbind-cn.org), which is also used as the data set in the CASF benchmark (http://www.pdbbind-cn.org/casf.asp), was employed. This data set includes 285 test cases, which are all known ligands docked into the holo-structures [13]. The PDB codes of all the test cases are listed in Table 1 and classified according to the number of torsions. Additionally, to evaluate the docking performance in apo structures, which is the more common scenario in real docking problems, a set of 24 human thrombin (PDB codes listed in Table 3) crystallographic complexes with known ligands were used in addition to the active-site ligand-free structures of thrombin [14, 15].

**Table 1:** Test cases classified by the number of torsions

Ntor^a^	PDB
**0**	3jya, 3udh, 4kzu
**1**	1bcu, 1gpn, 2xys, 3arv, 3rsx, 4ddk
**2**	1c5z, 1o5b, 1r5y, 1 s38, 2iwx, 2weg, 2yki, 3ary, 3g2z, 3gv9, 3kr8, 3pxf, 3twp, 3u5j, 3zt2, 4f09, 4gfm, 4jsz, 4 k77, 4kzq, 4mme, 4owm, 4u4s
**3**	1e66, 1gpk, 1qkt, 1uto, 1ydr, 2pog, 2wer, 3acw, 3ao4, 3bgz, 3g3l, 3gy4, 3kgp, 3pyy, 3wtj, 4cr9, 4de1, 4de3, 4e6q, 4ih5, 4j21, 4llx, 4m0y, 5c28
**4**	1q8u, 1syi, 2al5, 2cbv, 2hb1, 2j7h, 2v00, 2wcn, 2wtv, 2ymd, 3b27, 3dx1, 3f3a, 3f3c, 3g0w, 3 g2, 3lka, 3n7a, 3qqs, 3rr4, 3u8k, 3u8n, 4abg, 4ddh, 4dli, 4hge, 4ivb, 4j28
**5**	1nc3, 1o3f, 1oyt, 1p1n, 1p1q, 1 ps3, 1q8t, 2r9w, 2wn9, 2xj7, 3d4z, 3d6q, 3dd0, 3fcq, 3fur, 3gbb, 3jvr, 3qgy, 3rlr, 3ryj, 3uuo, 4e5w, 4f9w, 4gkm, 4ih7, 4ivc, 4 k18, 4kz6, 4m0z, 4mgd, 4pcs, 4qac, 5aba
**6**	1lowh, 1pxn, 1w4o, 2brb, 2c3i, 2j78, 2p15, 2qe4, 2xnb, 3dx2, 3dxg, 3e93, 3ebp, 3f3d, 3f3e, 3gc5, 3k5v, 3l7b, 3r88, 3syr, 3ui7, 3uo4, 4bkt, 4ivd, 4jxs, 4rfm, 4twp, 5dwr
**7**	1nc1, 1y6r, 1yc1, 1z9g, 2fvd, 2vvn, 2w66, 2wvt, 2 × 00, 2xbv, 2xii, 2zb1, 3b65, 3cj4, 3e92, 3ehy, 3fv2, 3g2n, 3nq9, 3up2, 4ciw, 4de2, 4djv, 4eor, 4f2w, 4j3l, 4jfs, 4jia, 4lzs
**8**	1bzc, 1sqa, 1ydt, 1z95, 2br1, 2cet, 2qbr, 2v7a, 2w4x, 2xb8, 2xdl, 2zcq, 2zy1, 3aru, 3b5r, 3fv1, 3ge7, 3ivg, 3jvs, 3mss, 3n76, 3n86, 3p5o, 4dld, 4qd6, 4wiv
**9**	4k1i, 1mq6, 1nvq, 1o0h, 2y5h, 2zda, 3e5a, 3gnw, 3u9q, 3wz8, 4cra, 4crc, 4f3c, 4ty7, 4w9i, 4x6p
**10**	1z6e, 2qbp, 2qnq, 3arq, 3ejr, 3nx7, 3oe4, 3ozt, 3zdg, 3zsx, 4w9c
**11**	1vso, 2qbq, 2vw5, 2wca, 2yfe, 2zcr, 3b68, 3nw9, 3oe5, 3ozs, 3ueu, 4agn, 4eky, 5c2h
**12**	2p4y, 2wbg, 3b1m, 4agp, 4agq, 4ea2, 4eo8
**13**	1 h22, 2fxs, 2yge, 3coz, 3kwa, 3myg, 3uev, 4cig, 4w9h, 5a7b
**14**	1g2k, 1lpg, 1qf1, 3coy, 4 g0
**15**	1a30, 1 h23, 3arp, 3tsk, 3uew, 3zso, 4ogj
**16**	1u1b, 3bv9, 3utu
**17**	1eby, 3o9i, 3pww, 3uex, 4tmn
**18**	5tmn
**19**	4w9l
**20**	2vkm
**23**	4gid
**24**	3prs
**33**	3uri
**36**	3ag9

^a^Number of torsions

### Hardware and experimental setting

The docking experiments were run on a personal computer with an Intel® i7–6850 core 3.60 GHz processor, 12-GB RAM and an Ubuntu 16.04 Linux platform. Based on the PDBbind coreset, the proposed DGLRDPSO algorithm and the compared algorithms, including LGA, LPSO, and LRDPSO, were all implemented in Autodock version 4.2.6.

In our experiments, the default docking configurations of Autodock 4.2.6 are used. The grid size was set to be 60 × 60 × 60 points with a spacing of 0.375 Å, which corresponds to a cube with an edge length of 22.5 Å; the search area of each test case was centred on the predicted binding site; the initial swarm size was set to 150; the total number for all the particles’ fitness to be evaluated was set to 2.5 × 10^6^, including the fitness evaluated in the search algorithms themselves and in the local search method. It should be noted that for all the compared algorithms, the components in all the dimensions of each particle’s position, which represent the translations, orientations, and torsion angles of the ligand, were all randomly initialized within the corresponding ranges. Each test case was docked 30 times by using all four tested algorithms.

The DGLRDPSO algorithm adopted the parameter settings mentioned in section 2. With respect to the other algorithms, in LGA, the crossover rate was set to 0.8 and the mutation rate was set to 0.02; in LPSO, the inertia weight decreased linearly from 0.9 to 0.4 and *c*_1_ = *c*_2_ = 2.05; in LRDPSO, *α* decreased linearly from 0.9 to 0.3 and *β* = 1.45. For the Solis and Wets method, all the parameters in these three algorithms were set in the same way as those in the DGLRDPSO (mentioned in section 2.4), except that in the LGA, there is a probability of 0.06 to perform a local search on each individual [5].

### Scoring function

In Autodock 3.05, an empirical binding free energy function has been used as the scoring function to estimate the docked energy of a docking conformation. The total docked energy of a candidate solution *X* can be expressed as:

15 $$ \mathit{\min}{E}_{total}(X)={E}_{vdw}+{E}_{hbond}+{E}_{elec}+{E}_{internal}+{E}_{desolvation} $$

where the first three terms are the van der Waals force, hydrogen bonding, and electrostatic potential, respectively. The sum of the three are the intermolecular energies. *E*_*internal*_ is the internal energy of the ligand, also containing the first three terms. *E*_*desolvation*_ models desolvation upon binding and the hydrophobic effect [3]. Thus, the docked energy is the sum of the intermolecular interaction energy between the ligand and the protein and the intramolecular interaction energy of the ligand. A detailed explanation of this function can be found in [5].

## Results and discussion

### Evaluation indexes

In this paper, the results of the different algorithms are compared in terms of three evaluation indexes, i.e., the final docked energy, the binding free energy and the root mean squared deviation (RMSD).

The docked energy is calculated by the scoring function during the whole searching process by equation (15), and the final docked energy directly reflects the performance of the search algorithms. A lower final docked energy corresponds to a better search performance of the algorithm.

The binding free energy is only reported at the end of docking, which is the sum of the intermolecular energy and the torsional free energy. In Autodock, this evaluation index is used to rank the final docking conformations, and its value is instructive for the selection and specific research of the final conformations. The binding free energy should be distinguished from the final docked energy because it does not include the internal or intramolecular interaction energy of the ligand. The reason for this is that this part of the energy cannot improve the accuracy of the binding free energy model, while for the docked energy, it truly affects the docking results [5].

RMSD is another frequently used index to evaluate docking conformations [20]. It is the displacement of atoms in a docked pose compared to the X-ray crystal protein-ligand structure (reference structure) and is calculated by

16 $$ rmsd=\sqrt{\frac{1}{N}\sum \limits_{i=1}^N{d}_i^2} $$

where *d*_*i*_ is the Euclidean distance between *N* pairs of equivalent atoms *i*. As a general criterion, an RMSD with no more than 2 Å for a docking result is usually considered to be successful [20]. In our experiments, this evaluation standard is also adopted. It should be noted that a lower docked energy or lower binding free energy does not always imply a better RMSD but only implies the higher possibility of a successful docking result.

### Search performance analysis

In this section, the final docked energy for all the test cases in PDBbind coreset v.2016 was compared among the different search algorithms since the final docked energy can directly demonstrate the performance of the search algorithms. Since there are 285 test cases tested by four algorithms, to show the results more clearly, the difference between the reference value and the result of the tested algorithm (*Dref*) is defined as

17 $$ Dref={Value}_{test}-{Value}_{ref} $$

where *Value*_*ref*_ and *Value*_*test*_ are the compared indicator value (e.g., mean value, best value) of the reference algorithm and the tested algorithm, respectively. In each test case, the reference algorithm is considered to have the lowest value of the compared indicator. Hence, the *Dref* value of the reference algorithm itself is obviously 0, and for the non-reference algorithms, a higher *Dref* of an algorithm corresponds to a worse algorithmic performance.

**Fig. 1:**
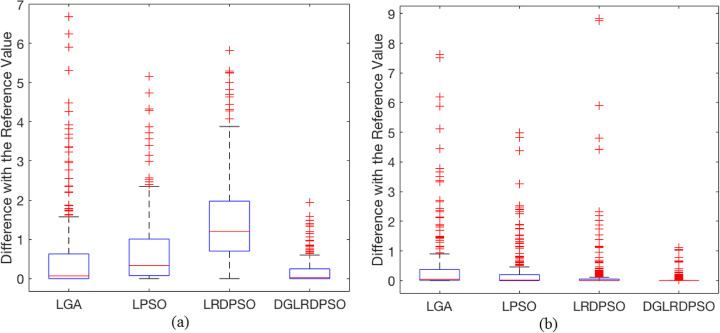
The box plots for the *Dref* results of the final docked energy. **a** The box plot for the *Dref* results of the mean final docked energy, (**b**) The box plot for the *Dref* results of the best final docked energy

With the above definition, the box plots of the *Dref* results, including the mean values and best values of the final docked energy for all the test cases in PDBbind coreset v.2016, are shown in Fig. 1a and Fig. 1b, respectively. Note that there are many test cases in PDBbind coreset v.2016, the specific *Dref* of the mean values and best values for the final docked energy thus cannot be illustrated in detail in this section, and plotting the *Dref* results in one figure can only provide the visualization of the difference between different search algorithms for all the docking test cases (both the *Dref* values for each test case and the plots for *Dref* results can be seen in Additional file 1). By contrast, the box plots in Fig. 1 show some statistical information of the *Dref* results, which can intuitively reflect the overall difference in the final docked energy results of all 285 docking test cases between different search algorithms.

In both Fig. 1a and Fig. 1b, the interquartile range (the height of the box), the medium value, and the maximum value (the minimum value for all algorithms is 0) of the DGLRDPSO algorithm are all close to 0 and much smaller than those of the other algorithms, especially for the *Dref* of the best values. This means that in most test cases, DGLRDPSO can (or nearly) obtain the best results for both the mean values and the best values of the final docked energy among all the algorithms. Moreover, for Fig. 1a, most of the outliers for the *Dref* of the DGLRDPSO are concentrated near its maximum values, but they are even smaller than the maximum values of the other three algorithms. This phenomenon, along with the small interquartile range of DGLRDPSO in Fig. 1a, demonstrates that the robustness of the proposed algorithm is much higher than that of the other three algorithms. With respect to the *Dref* of the best values, the advantage of the DGLRDPSO over the other algorithms is more remarkable than the *Dref* of the mean values according to Fig. 1b, implying that the DGLRDPSO performs better in finding results with higher quality than all the other algorithms. In addition, by comparing LRDPSO with the proposed algorithm, we find that most of the *Dref* values of the best results obtained by LRDPSO are comparable to those obtained by DGLRDPSO, except for several outliers with relatively large values. However, its performance shown in Fig. 1a is almost the worst among all the compared algorithms, which indicates that the 2PDC strategy can significantly improve the robustness of the proposed algorithm and further increase the probability for DGLRDPSO to find a lower energy than LRDPSO within a limited number of trails.

**Fig. 2:**
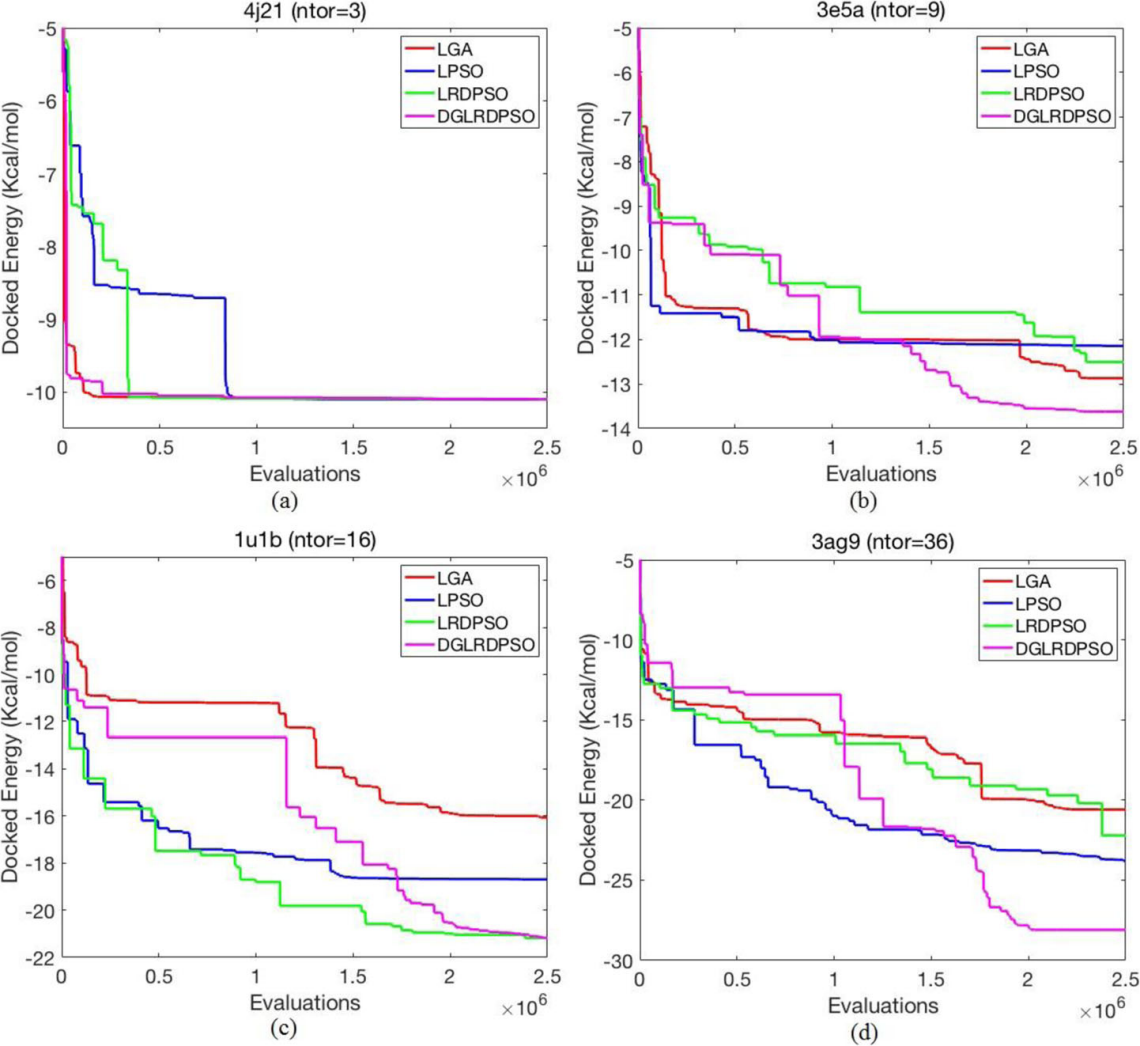
Convergence performance of the lowest final docked energy for four selected test cases during the evolving process. **a** The convergence performance comparison for 4j21, (**b**) The convergence performance comparison for 3e5a, (**c**) The convergence performance comparison for 1u1b, (**d**) The convergence performance comparison for 3ag9

To further analyse the search performance of the proposed algorithm, we plot, in Fig. 2, the convergence performance of the lowest final docked energy for four selected test cases. The curves for each search algorithm in Fig. 2 are plotted by recording the docked energy (e.g., the fitness value of the historical best particle in the current iteration) in each iteration during the whole search process. Due to space limitations, we cannot plot the convergence performance for all 285 test cases, and thus, we selected the four test cases that cover the different numbers of torsions to observe the convergence performance for the different docking problems whose search dimensionalities range from low to high. As the figure shows, when there are only a few torsions in the test case (e.g., 4j21), the DGLRDPSO algorithm can quickly find a relatively low energy, and its final docked energy is comparable to those of other algorithms; when the number of torsions becomes higher, DGLRDPSO converges much more slowly, sometime failing to find a low enough energy at the beginning but overcoming the energy barrier during the evolving process and thus obtaining a much better result than the other algorithms at the end. It should be noted that although LRDPSO can occasionally find a final docked energy equivalent to that found by the DGLRDPSO in highly flexible ligand docking problems (e.g., 1u1b), it cannot perform well in all test cases (e.g., 3e5a and 3ag9) due to its poor robustness, which further demonstrates the effectiveness of the 2PDC strategy.

Referring to the box plots of the *Dref* results and the specific analysis of the convergence performance for several test cases, we can conclude that DGLRDPSO has the best performance and robustness among all the compared algorithms. In addition, these results also illustrate that DGLRDPSO performs much better than LRDPSO in most cases, especially in terms of robustness. Therefore, we further evaluated only LGA, LPSO and DGLRDPSO by comparing their docking performance in the following sections.

### Holo-structure docking analysis

**Fig. 3:**
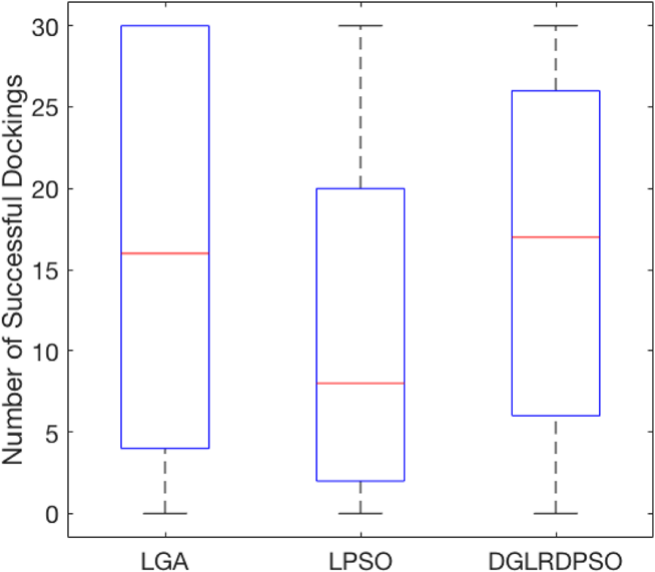
The box plot for the number of successful docking results (RMSD ≤ 2 Å)

To evaluate the docking accuracy of the different algorithms, Fig. 3 illustrates the box plot for the number of successful dockings (with an RMSD of no more than 2 Å) out of 30 runs for LGA, LPSO and DGLRDPSO (the overview plot for this criterion is shown in Additional file 1). Note that Fig. 3 is plotted according to the successful docking numbers and not by the *Dref* values, since the real successful docking rate of each search algorithm, which is an important criterion to evaluate the docking accuracy, cannot be illustrated by the *Dref* values. However, the *Dref* values of the successful docking numbers for each test case can be accessed in Additional file 1.

According to Fig. 3, the interquartile range of the DGLRDPSO is the smallest among the three algorithms, which also demonstrates the robustness of the proposed algorithm. The LPSO, as shown by all the quartile lines in Fig. 3, has the worst performance compared to the other two algorithms in most cases in terms of the successful docking numbers. For LGA, the upper quartile line in Fig. 3 is 30, which is higher than that of DGLRDPSO and is much higher than that of LPSO, meaning that LGA can find 30 or nearly 30 successful docking conformations in more test cases than the other two algorithms. More specifically, comparing the results of the successful docking numbers of LGA and DGLRDPSO in Additional file 1, we can find that LGA is able to obtain a large number of successful dockings for many less flexible ligand test cases (torsions < 9). However, for highly flexible ligand ones (torsions ≥ 9), LGA has no significant advantage over DGLRDPSO. Thus, the reason why the statistical results of LGA are better than those of DGLRDPSO in terms of successful docking numbers in Fig. 3 is conjectured to be that the low-torsion test cases account for the majority of the PDBbind coreset v.2016. To justify this, Table 2 lists some statistics of the RMSD results for the three compared algorithms. According to Table 2, it is clear that for the less flexible ligand docking problems, the average successful docking number of LGA is larger than those of the other two algorithms, although the result of DGLRDPSO is close to that of LGA. Meanwhile, for the highly flexible ligand test cases, the advantage of DGLRDPSO over the others is more significant, which definitely verifies the aforementioned conjecture. Nevertheless, DGLRDPSO is still considered to have the best performance in terms of the RMSD-related results among all the compared algorithms, since it can obtain the best results for four terms, as shown in Table 2, and for the other two terms, there are insignificant differences between the results of DGLRDPSO and the results of the best algorithms.

**Table 2:** Statistics of the RMSD results for LGA, LPSO and DGLRDPSO

	LGA	LPSO	DGL RDPSO
**Average successful docking number for all test cases**	**16.2**	11.09	15.93
**Average successful docking number for less flexible ligand test cases (torsions < 9)**	**19.4**	12.43	18.0
**Average successful docking number for highly flexible ligand test cases (torsions** ≥ **9)**	8.84	7.95	**11.06**
**Number of failed docking test cases**	36	43	**29**
**Average of RMSD’s Mean Values for all test cases (Å)**	2.41	2.87	**2.26**
**Average of RMSD’s Best Values for all test cases (Å)**	1.20	1.17	**1.10**

**Fig. 4:**
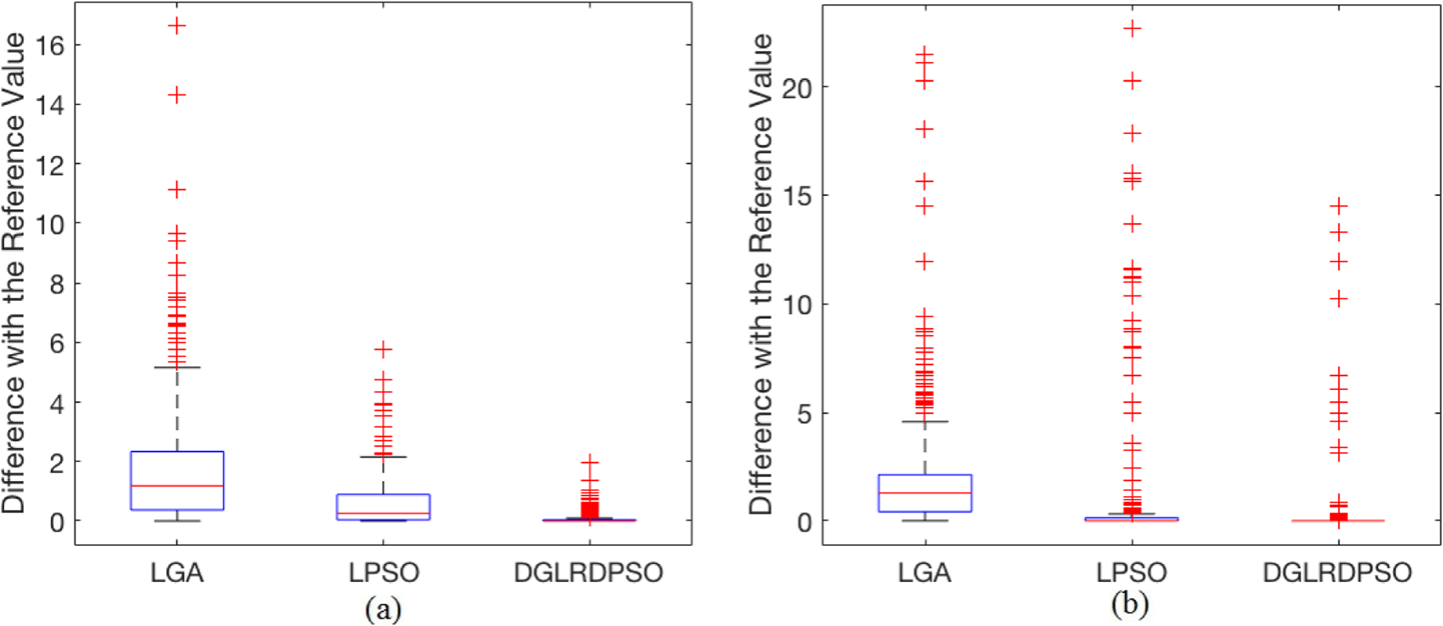
The box plot for the *Dref* results of the binding free energy. **a** The box plot for the *Dref* of the mean binding free energy, (**b**) The box plot for the *Dref* of the lowest binding free energy among all the successful docking conformations

Figure 4a shows the statistical results of the *Dref* for the mean values of the binding free energy. The robustness of DGLRDPSO is found to be the best among all the compared algorithms, which is similar to the results obtained by the final docked energy comparison analysis. In most cases, the DGLRDPSO algorithm can obtain the best mean binding free energy, since all the quartile lines of DGLRDPSO in Fig. 4a are close or equal to 0. Unlike the results for the mean final docked energy, LPSO shows better robustness and performance than LGA in terms of the mean binding free energy, for according to the specific *Dref* values in Additional file 1, the difference between the mean energy of LGA and that of the reference one becomes larger as the number of torsions increases.

In addition to the *Dref* of the mean binding free energy, the box plot for the *Dref* of the lowest binding free energy among all the successful docking conformations (hereinafter called the lowest successful binding free energy) is illustrated in Fig. 4b. It should be noted that if the RMSDs of the final docking conformations found by an algorithm for a test case are all larger than 2 Å (the number of such test cases for each algorithm is recorded in Table 2), the corresponding lowest successful binding free energy is recorded as 0, and the corresponding *Dref* is thus the inverse of the reference energy value. According to Fig. 4b, the interquartile range, the medium value, and the maximum value of the LGA algorithm are the worst among all the compared algorithms, which is similar to its performance in the mean binding free energy, implying that the LGA algorithm does not perform well in finding a conformation with a lower binding free energy in most cases compared to the other two algorithms. For LPSO, although the interquartile range and the maximum value are close to 0, the number of outliers shown in Fig. 4b is more than that of DGLRDPSO, which means that it shows worse robustness than DGLRDPSO in terms of the lowest successful binding free energy. The maximum and minimum values of DGLRDPSO in Fig. 4b are both 0, which means that in most test cases, DGLRDPSO can obtain the best result in terms of the lowest successful binding free energy. The outliers of DGLRDPSO with relatively high values in Fig. 4b represent test cases in which the proposed algorithm failed to find one successful docking conformation (see statistical results in Additional file 1).

Table 3 lists some statistical results of the conformations with the lowest binding free energy and the lowest successful binding free energy for the three compared algorithms. It should be pointed out that if the search algorithm failed to find a successful docking conformation, its rank of the conformation with the lowest successful binding free energy is recorded as 31, 1 larger than the number of trials. According to Table 3, in all three terms, DGLRDPSO is the best among the three algorithms. This means that for the proposed algorithm, the conformations with the lowest successful binding free energy have the best average ranks, and in many cases, these conformations are top-scored conformations. Thus, it can be concluded that DGLRDPSO has the best performance in finding a correct conformation with a low enough binding free energy among all search methods, since once DGLRDPSO can find one or more successful docking conformations, it is generally able to find the successful docking conformation with the highest rank and lowest binding free energy among all the compared algorithms.

**Table 3:** Statistics of the lowest-energy-related conformations for LGA, LPSO and DGLRDPSO

	LGA	LPSO	DGL RDPSO
**Number of test cases which can identify the correct binding conformation with the lowest binding free energy as the criterion**	181	187	**195**
**Average RMSD of the conformations with the lowest binding free energy for all test cases (Å)**	2.10	2.05	**1.93**
**Average rank of the conformations with the lowest successful binding free energy for all test cases**	7.54	8.14	**6.42**

### Apo-structure docking analysis

As the test cases in PDBbind coreset v.2016 are all known ligands docked to holo-structures, 24 ligands were docked to the active site of the apo structures of the human thrombin (PDB code 1vr1) using flexible ligand docking methods. Table 4 lists the information and the statistical results for all these test cases docked by LGA, LPSO and DGLRDPSO.

**Table 4:** Statistical results for the apo-structure docking conformations found by LGA, LPSO and DGLRDPSO

PDB^**a**^	Ntor^**b**^	LGA			LPSO			DGLRDPSO		
		NS^**c**^	ME^**d**^	LE^**e**^	NS	ME	LE	NS	ME	LE
1bcu	2	0	**−3.09**	–	0	−3.01	–	0	−2.77	–
1aht	7	0	−3.24	–	0	−5.05	–	0	**−5.13**	–
1ay6	9	1	−7.42	−8.51	0	−9.18	–	**3**	**−9.38**	**−10.59**
1bhx	9	**1**	−6.90	**−8.04**	0	−11.61	–	0	**−11.82**	–
1tom	9	5	−8.17	−9.17	**18**	−11.36	**−12.39**	15	**−11.49**	−11.86
1uma	9	0	−3.60	–	0	−6.36	–	0	**−7.18**	–
1afe	10	0	−6.02	–	1	**−9.64**	**−9.76**	2	−9.48	− 9.75
1bb0	10	**5**	−7.46	−8.86	2	− 12.47	−13.89	4	**−12.80**	**−14.00**
1a61	12	0	−7.14	–	0	−12.92	–	0	**−13.47**	–
1b5g	12	0	−7.57	–	**1**	−12.66	−11.59	**1**	**−12.87**	**−12.36**
1ba8	12	0	−5.62	–	0	−11.76	–	**2**	**−11.80**	**−13.57**
1a3b	13	0	−3.63	–	0	−8.48	–	0	**−8.68**	–
1fpc	13	0	−6.94	–	**4**	**−11.62**	**−12.09**	3	−11.42	−11.67
1tbz	13	1	−7.41	−9.81	2	−13.95	−13.90	**4**	**− 13.95**	**−14.84**
1a46	14	**4**	−7.35	−9.85	**4**	−14.23	−15.37	1	**−14.53**	**−15.87**
1ae8	14	2	−5.58	−6.88	14	−10.82	−11.17	**17**	**−11.10**	**−11.63**
1lhc	14	1	−6.47	−8.80	0	−11.76	–	**2**	**−12.01**	**−12.89**
1lhg	14	3	−5.26	−6.95	**8**	−10.77	−12.13	7	**−11.00**	**−12.25**
1a4w	15	0	−5.99	–	0	−13.68	–	**1**	**−13.88**	**−14.69**
1a5g	15	**1**	−7.19	−7.94	0	−13.02	–	**1**	**−13.37**	**−15.05**
1lhd	15	3	−5.47	−7.09	4	−10.79	−12.04	**9**	**−11.15**	**− 12.27**
1aix	16	1	−4.74	−6.15	**2**	**−11.76**	−11.70	**2**	−11.62	**−12.25**
1lhe	16	0	−4.30	–	1	−11.47	**−12.39**	**2**	**−11.58**	−12.26
1awf	25	0	2.56	–	0	−6.06	–	0	**−6.42**	–
***Wins***		*4*	*1*	*1*	*6*	*3*	*4*	***11***	***20***	***13***

^a^The PDB code of the thrombin crystallographic complex

^b^The number of torsions of the corresponding ligand

^c^The number of successful dockings

^d^The mean binding free energy for 30 final docking conformations

^e^The lowest binding free energy among all the successful docking conformations

**Fig. 5:**
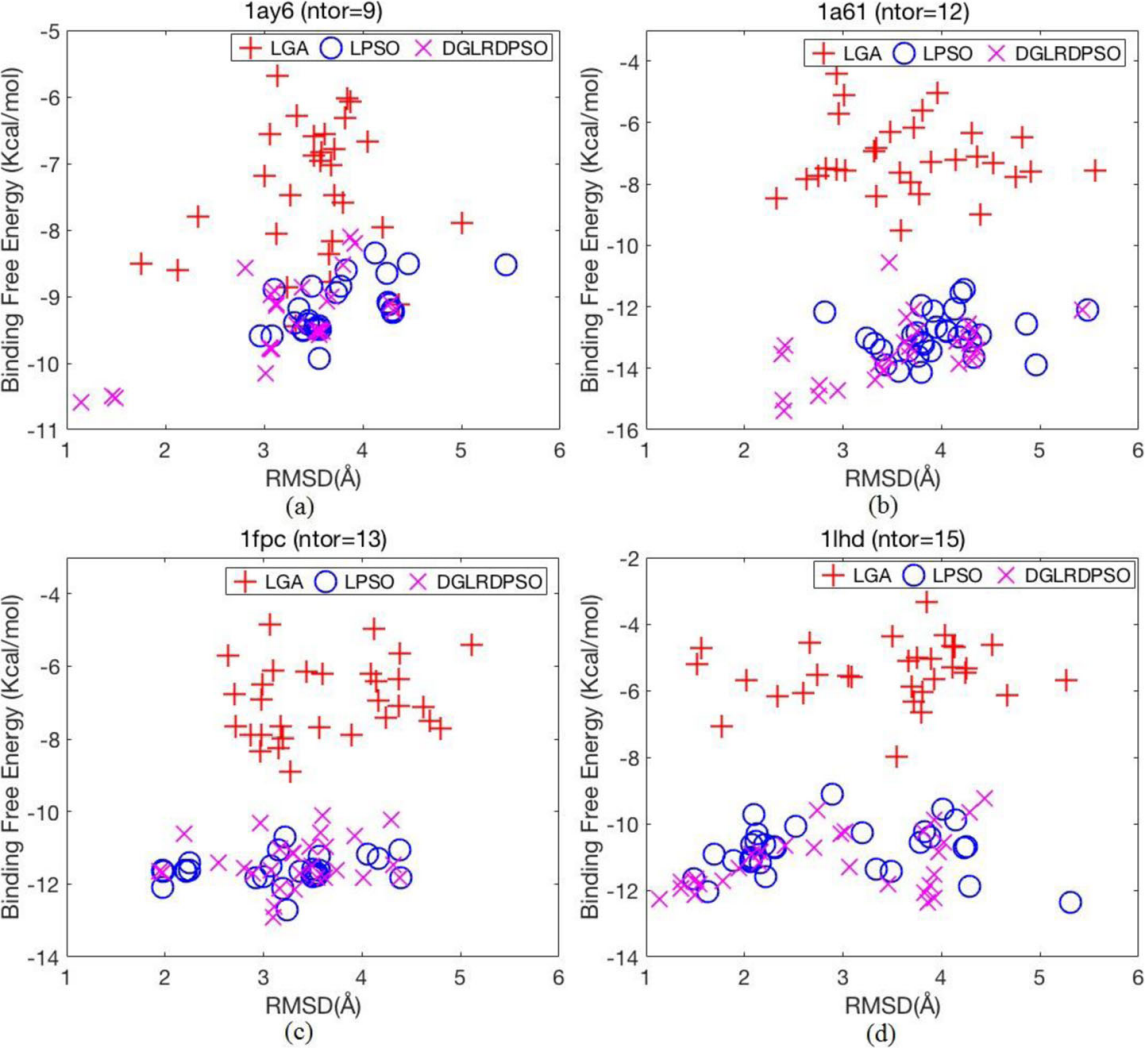
Energy versus RMSD obtained by LGA, LPSO, and DGLRDPSO. **a** Energy versus RMSD plot for 1ay6, (**b**) Energy versus RMSD plot for 1a61, (**c**) Energy versus RMSD plot for 1fpc, (**d**) Energy versus RMSD plot for 1lhd

Similar to the analysis for the holo-structure docking in the last section, Table 4 also records the number of successful dockings, the mean binding free energy, and the lowest successful binding free energy obtained by the three compared algorithms for all test cases. In addition, Fig. 5 shows the energy versus RMSD plots for the four selected test cases to compare the docking performance of LGA, LPSO and DGLRDPSO more specifically. The four selected test cases in Fig. 5 are representative of all of the test cases. For 1ay6, LGA and DGLRDPSO were able to find successful docking conformations, and for 1a61, all the compared algorithms failed. LPSO and DGLRDPSO generated successful docking for 1fpc, and all the compared algorithms were successful for 1lhd. The energy versus RMSD plots for the other test cases are not shown in this paper because of space limitations.

The “*Wins*” results in Table 4 show that the DGLRDPSO algorithm performs the best among all algorithms in all three terms, and the distributions of points representing the conformations generated by the DGLRDPSO in Fig. 5 further demonstrate that the proposed algorithm has a higher probability of finding the conformations with both lower binding free energy and smaller RMSD in most cases. Table 4 shows that, in many test cases, LGA failed to find a successful docking conformation within 30 trials. Moreover, the energy-related results in Table 4 and all the plots in Fig. 5 reveal that the energy of the conformations found by LGA is much worse than those found by the other two algorithms. This demonstrates that LGA can hardly obtain a low-energy result just as it performs in the holo-structure test cases. With respect to the LPSO, its mean binding free energy is comparable to that of the DGLRDPSO and performs better in terms of low-energy conformations in some test cases (see 1fpc in Fig. 5). However, in most of the test cases, DGLRDPSO can still find conformations with much lower energy in some trials and thus obtains lower RMSDs (see 1ay6, 1a61, and 1lhd in Fig. 5), since DGLRDPSO has a stronger ability to overcome the energy barriers than LPSO. Even in those test cases in which no algorithm can find one successful docking conformation, DGLRDPSO also has the best performance so that it can find the conformations closest to the reference conformation (e.g., 1a61).

According to the aforementioned analysis, it can be concluded that DGLRDPSO is generally superior to LGA and LPSO in terms of binding free energy and RMSD, especially for highly flexible ligand docking problems, and its robustness is the best among the compared methods. However, the efficiency of these docking methods should be taken into consideration, since it is not worth consuming too much computational time to obtain better docking performance and robustness of the algorithm in some real docking applications, e.g., virtual screening [2]. Hence, Table 5 lists the average docking time for each holo-structure (test cases in Table 1) and apo-structure (test cases in Table 4) test case over 30 runs performed by each of the three compared algorithms. The individual docking times for each test case performed by each of the compared algorithms are recorded in Additional file 2. Table 5 shows that DGLRDPSO is the most efficient algorithm among all methods in terms of the average docking time and the average *Dref* values for docking time.

**Table 5:** Statistics of the docking time for all test cases taken by LGA, LPSO and DGLRDPSO

	LGA	LPSO	DGLRDPSO
**Average docking time of each test case**	4992 s	4637 s	**4582 s**
**The average*Dref*value for the docking time of each test case**	680 s	326 s	**270 s**

## Conclusions

Flexible ligand docking is one the most frequently used methods in protein-ligand docking. To solve this problem, heuristic algorithms are popular and effective methods for finding the suitable sites and conformations of the ligands. In this paper, based on the Autodock software package and its scoring function, a novel algorithm called diversity-guided Lamarckian random drift particle swarm optimization (DGLRDPSO) is proposed. In this algorithm, the 2PDC diversity control strategy and the local search method are used to further improve the performance and robustness of the RDPSO algorithm. The simulation results from the comparison among LGA, LPSO and LRDPSO on PDBbind coreset v.2016 show that the 2PDC strategy gives the proposed algorithm generally better performance and robustness than LPSO and LGA and LPSO, as shown by the mean and best final docked energy. With respect to the docking performance, DGLRDPSO can generally find better docking conformations than LGA and LPSO in terms of the binding free energy and RMSD for both holo- and apo-structure docking test cases. In particular, its advantage over LGA in terms of RMSD is more remarkable for highly flexible ligand docking problems than for less flexible ones. Moreover, DGLRDPSO shows better robustness than the other compared algorithms in terms of the number of torsions. Therefore, the proposed DGLRDPSO algorithm is expected to be a reliable choice for flexible ligand docking in Autodock software.

Our future research will focus on further modification of the proposed algorithm to make it applicable for other complicated docking problems, such as docking with side-chain flexibility and blind docking.

## Supplementary information

**Additional file 1.** Docking performance statistics of each holo-structure test case for all tested algorithms. The additional file 1 includes three tables that contain the statistics of the final docked energy, binding free energy and RMSD results for the holo-structure docking results of each test case.

**Additional file 2.** Docking time expense of all the test cases for LGA, LPSO and DGLRDPSO. The additional file 2 includes one table that contains the docking time expense of all the holo- and apo-structure docking test cases for all the related tested algorithms.

## Data Availability

The datasets generated and analysed during the current study are available in the PDBbind coreset repository, http://www.pdbbind-cn.org/casf.asp, and Protein Data Bank repository, https://www.rcsb.org.

## Abbreviations

RDPSO: Random Drift Particle Swarm Optimization
DGLRDPSO: Diversity-guided Lamarckian Random Drift Particle Swarm Optimization
2PDC: Two-phased diversity control
LGA: Lamarckian genetic algorithm
LPSO: Lamarckian particle swarm optimization
LRDPSO: Lamarckian random drift particle swarm optimization
RMSD: Root mean squared deviation
PSO: Particle swarm optimization
pbest: personal best
gbest: global best
mbest: mean best
